# Fetomaternal Outcome Among Antenatal Attendees With Advanced Maternal Age: A Cohort Study

**DOI:** 10.7759/cureus.99390

**Published:** 2025-12-16

**Authors:** Hina Toufique, Saira Bano, Afsheen Shahid Shaikh, Shaista Rafique, Lamia Rafique, Iman Zishan

**Affiliations:** 1 Obstetrics and Gynaecology, Al Musanaah Extended Health Center, Muladdah, OMN; 2 Obstetrics and Gynaecology, Rustaq Hospital, Al-Rustaq, OMN; 3 Obstetrics and Gynaecology, Tbilisi State Medical University, Tbilisi, GEO

**Keywords:** advanced maternal age, antenatal care, fetal outcomes, maternal outcomes, oman

## Abstract

Background

Advanced maternal age (AMA) has become increasingly prevalent as women postpone childbearing due to social, cultural, and economic reasons. Previous studies have linked AMA to a higher risk of adverse maternal and fetal outcomes, but regional data from Oman remain limited.

Objective

This study aimed to evaluate the association between AMA and pregnancy outcomes in women attending antenatal visits at Musannah Polyclinic and AR-Rustaq General Hospital.

Methodology

This observational comparative cohort study was conducted at Musannah Polyclinic, Sultanate of Oman, over 12 months beginning in July 2024. A total of 646 pregnant women were enrolled, comprising 323 women aged ≥40 years (AMA group) and 323 women <35 years (control group). Participants were recruited using non-probability convenience sampling. Data collection included demographic variables, maternal comorbidities, pregnancy complications, and fetal outcomes. Laboratory and imaging assessments were performed according to Ministry of Health protocols. Statistical analysis was conducted using SPSS version 26, with t-tests and chi-square tests applied for group comparisons, and logistic regression performed to identify independent predictors of adverse outcomes.

Results

The mean age in the AMA group was 42.8 ± 2.1 years versus 28.6 ± 3.5 years in controls (p < 0.001). AMA women had significantly lower spontaneous vaginal delivery rates (52/323; 16.1% vs. 149/323; 46.1%, p < 0.001) and higher preterm delivery rates (14/323; 4.3% vs. 5/323; 1.5%, p = 0.03). Gestational diabetes was significantly higher in AMA women (156/323; 48.3% vs. 47/323; 14.6%, p < 0.001), as were hypertensive disorders (38/323; 11.8% vs. 9/323; 2.8%, p < 0.001) and thyroid disorders (38/323; 11.8% vs. 11/323; 3.4%, p < 0.001). Fetal complications were also increased, including miscarriage (17/323; 5.3% vs. 4/323; 1.2%, p = 0.004) and NICU admissions (11/323; 3.4% vs. 3/323; 0.9%, p = 0.04). Logistic regression confirmed AMA as an independent predictor of gestational diabetes, hypertensive disorders, cesarean delivery, miscarriage, NICU admission, and thyroid disorders.

Conclusion

AMA is strongly associated with adverse maternal and fetal outcomes, underscoring the need for targeted antenatal monitoring, early screening, and specialized counseling in this high-risk group.

## Introduction

An increasing number of women in developing countries are postponing pregnancy [1]. Previous studies have shown associations between advanced maternal age (AMA) and a higher risk of miscarriage and chromosomal abnormalities of the fetus [2,3], whereas studies on congenital malformations, preterm birth, and stillbirth have reported conflicting findings [3,4]. Many earlier studies were inadequately powered to explore associations with rare adverse outcomes or to adjust for important confounders. Furthermore, most studies investigating rare fetal outcomes have based their results on adverse outcome prevalence at 20-40 weeks of gestation or live birth prevalence [5,6], thereby excluding anomalies that may lead to fetal loss before 20 weeks. Additionally, earlier studies focused on selected groups, such as women undergoing invasive testing or those accessing private antenatal care, which may introduce bias and limit generalizability.

Globally, women are increasingly delaying childbearing into their late 30s and 40s [7]. This trend is largely attributed to career aspirations, equal opportunities in the job market, and the desire for financial stability [5]. In low-resource settings, advanced-age pregnancy is often more common among multiparous women due to ineffective family planning and a cultural preference for larger families [8]. Although many authors define AMA as pregnancy at 35 years and above [3], in the medical literature it is frequently defined as age over 40 years [1]. Most studies agree that pregnancy at AMA is rarely without risks; however, conclusions based on individual obstetric or perinatal outcomes remain inconsistent. Some reports indicate no significant differences between AMA and non-AMA pregnancies in terms of preterm birth, low birth weight, small for gestational age, or perinatal mortality [4], while others emphasize increased risks. The reasons for worse outcomes in AMA pregnancies remain debated. Some argue that maternal age alone is not an independent risk factor [8], while others suggest that adequate antenatal care and proper obstetric management may reduce risks and yield outcomes similar to those of younger women [2]. In contrast, Frick AP [4] found more adverse outcomes even among low-risk AMA women, indicating that age-related physiological changes may contribute beyond pre-existing disease.

Given these controversies and the limited regional data, it is important to investigate the impact of AMA in diverse populations. The rationale of this study is to evaluate maternal and fetal outcomes among women aged 40 years and above compared with younger women in a real-world antenatal setting in Oman. The objective is to examine the association between AMA and adverse pregnancy outcomes in pregnant women attending Musanaah Polyclinic and Rustaq Hospital.

## Materials and methods

This study was conducted at the Musannah Polyclinic and Rustaq Hospital, Sultanate of Oman, under the Ministry of Health, Directorate General of Health Services, South Batinah Governorate, Directorate of Planning and Studies. The study duration was 12 months, commencing on 16 July 2024. Ethical approval was obtained from the Research and Ethics Committee, South Batinah Governorate, with the approval code 03062024.

The study design was observational and analytical in nature, structured as a comparative cohort study. A total of 646 pregnant women were recruited and divided into two groups. The study group comprised 323 women of advanced maternal age, defined as 40 years and above, while the control group included 323 pregnant women below the age of 35 years. The sample size was calculated using the OpenEpi sample size calculator with a 95% confidence interval and 80% power, based on previously reported proportions of adverse pregnancy outcomes in advanced maternal age women compared to younger women. To ensure representativeness, the sample size was inflated to 323 per group.

Non-probability convenience sampling was employed to recruit participants among women presenting for antenatal visits at Musannah Polyclinic during the study period. Inclusion criteria were confirmed pregnancy with gestational age less than 20 weeks at the time of recruitment, willingness to participate, and regular follow-up at the study site. Women with known chronic medical illnesses such as pre-existing diabetes mellitus, chronic hypertension, renal disease, autoimmune disorders, or previous uterine surgery were excluded to minimize confounding variables that might independently influence pregnancy outcomes.

Detailed demographic and clinical information was collected from each participant through structured interviews and review of antenatal records. Maternal characteristics including age, gravidity, parity, BMI, educational status, and socioeconomic background were documented. Clinical parameters such as baseline blood pressure, hemoglobin levels, and random blood glucose were measured at enrollment. Throughout the pregnancy, maternal complications including gestational diabetes mellitus, hypertensive disorders of pregnancy, preeclampsia, antepartum hemorrhage, premature rupture of membranes, and preterm labor were carefully monitored and recorded. Fetal outcomes were assessed in terms of intrauterine growth restriction (IUGR), congenital anomalies, stillbirth, mode of delivery, Apgar scores, NICU admission, and neonatal mortality.

All laboratory investigations were performed in the Musannah Polyclinic laboratory using standard techniques according to Ministry of Health protocols. Ultrasound scans were carried out by qualified radiologists for fetal biometry and anomaly screening. Blood pressure was measured using a calibrated sphygmomanometer in the seated position after a minimum rest of 5 minutes. Gestational diabetes was diagnosed using the oral glucose tolerance test (OGTT) according to WHO guidelines. Hemoglobin concentration was determined via automated hematology analyzers to detect anemia.

Statistical analysis was carried out using the SPSS version 26. Continuous variables such as maternal age, hemoglobin level, BMI, and birth weight were expressed as mean ± SD, while categorical variables such as parity, presence of gestational diabetes, mode of delivery, and NICU admission were expressed as frequencies and percentages. Comparisons between the advanced maternal age group and the control group were made using the independent sample t-test for continuous variables and the chi-square test for categorical variables. Logistic regression analysis was performed to identify independent predictors of adverse maternal and neonatal outcomes after adjusting for potential confounders. A p-value of <0.05 was considered statistically significant.

## Results

A total of 646 pregnant women were enrolled in the study, comprising 323 women of advanced maternal age (≥40 years) and 323 women in the control group (<35 years). The mean age of participants in the advanced maternal age group was 42.8 ± 2.1 years, while the mean age of the control group was 28.6 ± 3.5 years (p < 0.001).

Demographic characteristics

Table 1 presents the age distribution of women in the advanced maternal age group. The majority, 271 (83.9%), were between 40 and 44 years, while 51 (15.8%) were between 45 and 48 years, and only one participant was above 49 years. Educational status was higher among women in the control group compared to advanced maternal age women, although the difference was not statistically significant (p = 0.08). Employment status, however, was significantly associated with maternal age, with fewer professionals among older mothers (p < 0.05).

**Table 1:** Demographic characteristics of study participants. *Significant at p < 0.05.

Variable	Advanced maternal age (n = 323)	Control (<35 years) (n = 323)	p-value
Mean age (years ± SD)	42.8 ± 2.1	28.6 ± 3.5	<0.001*
Age groups (years)			
25-29	-	184 (56.9%)	<0.001*
30-34	-	139 (43.1%)	
40-44	271 (83.9%)	-	
45-48	51 (15.8%)	-	
>49	1 (0.3%)	-	
Educational status			
Qualified	226 (70.0%)	254 (78.6%)	0.08
Unqualified	97 (30.0%)	69 (21.4%)	
Profession			
Professional	129 (40.0%)	176 (54.5%)	0.02*
Non-professional	194 (60.0%)	147 (45.5%)	

Maternal outcomes

Mode of delivery was significantly different between the two groups (p < 0.001). Women of advanced maternal age had a higher rate of lower segment cesarean section (LSCS), while spontaneous vaginal delivery (SVD) was more frequent in younger women. Preterm delivery (<37 weeks) occurred in 4.3% of older women compared to 1.5% in the control group. Hypertensive disorders and gestational diabetes mellitus (GDM) were also markedly higher among older women (p < 0.001) (Table 2).

**Table 2:** Maternal outcomes in advanced maternal age and control groups. SVD: Spontaneous vaginal delivery; LSCS: Lower segment cesarean section; IUGR: Intrauterine growth restriction; GDM: Gestational diabetes mellitus; PPH: Postpartum hemorrhage. *p < 0.05 considered statistically significant.

Maternal outcome	Advanced maternal age (n = 323)	Control (<35 years) (n = 323)	p-value
SVD	52 (16.1%)	149 (46.1%)	<0.001*
LSCS	42 (13.0%)	52 (16.1%)	0.21
Preterm delivery (<37 weeks)	14 (4.3%)	5 (1.5%)	0.03*
IUGR	2 (0.6%)	1 (0.3%)	0.56
Hypertensive disorders	38 (11.8%)	9 (2.8%)	<0.001*
Pre-eclampsia	1 (0.3%)	0 (0.0%)	0.31
GDM	156 (48.3%)	47 (14.6%)	<0.001*
Pre-existing diabetes	18 (5.6%)	3 (0.9%)	<0.001*
Thyroid disorders	38 (11.8%)	11 (3.4%)	<0.001*
PPH	3 (0.9%)	1 (0.3%)	0.31
ICU admission	1 (0.3%)	0 (0.0%)	0.31

Fetal outcomes

Adverse fetal outcomes were more frequent in the advanced maternal age group compared with controls. Miscarriage was significantly more common (5.3% vs. 1.2%, p < 0.01). Similarly, congenital anomalies, including Down syndrome and multiple anomalies, were noted exclusively in older mothers. NICU admissions were also significantly higher in this group (p < 0.05) (Table 3).

**Table 3:** Fetal outcomes in advanced maternal age and control groups. *Significant at p < 0.05. IUGR: Intrauterine growth restriction.

Fetal outcome	Advanced maternal age (n = 323)	Control (<35 years) (n = 323)	p-value
Miscarriage	17 (5.3%)	4 (1.2%)	0.004*
IUGR	2 (0.6%)	1 (0.3%)	0.56
Down syndrome	2 (0.6%)	0 (0.0%)	0.16
Multiple anomalies	2 (0.6%)	0 (0.0%)	0.16
Placenta praevia	1 (0.3%)	0 (0.0%)	0.31
NICU admission	11 (3.4%)	3 (0.9%)	0.04*
Neonatal mortality	2 (0.6%)	0 (0.0%)	0.16

Logistic regression analysis

Multivariate logistic regression analysis was performed to identify independent predictors of adverse pregnancy outcomes after adjusting for potential confounders including BMI, parity, and socioeconomic status. The results demonstrated that advanced maternal age (≥40 years) was an independent predictor of GDM, hypertensive disorders, cesarean delivery, miscarriage, and NICU admission. The adjusted odds ratios (aOR) with 95% CIs are presented in Table 4.

**Table 4:** Logistic regression analysis of predictors of adverse maternal and fetal outcomes. *Significant at p < 0.05.

Outcome	Predictor	Adjusted odds ratio (aOR)	95% CI	p-value
Gestational diabetes mellitus	Advanced age ≥40 years	4.72	3.26-6.82	<0.001*
Hypertensive disorders	Advanced age ≥40 years	3.89	1.92-7.86	<0.001*
Cesarean delivery	Advanced age ≥40 years	2.15	1.42-3.27	<0.001*
Miscarriage	Advanced age ≥40 years	3.41	1.13-10.29	0.03*
NICU admission	Advanced age ≥40 years	2.87	1.01-8.17	0.04*
Preterm delivery (<37 weeks)	Advanced age ≥40 years	1.92	0.82-4.46	0.12 (NS)
Thyroid disorders	Advanced age ≥40 years	2.74	1.36-5.53	0.004*

## Discussion

This study investigated the association between AMA and pregnancy outcomes among women attending antenatal clinics at Musannah Polyclinic. Our findings demonstrate that pregnancies in women aged 40 years and older are associated with increased risks of adverse maternal and fetal outcomes compared with younger mothers, consistent with a growing body of knowledge internationally.

In our cohort, the mean maternal age in the AMA group was 42.8 ± 2.1 years, significantly higher than in the control group (28.6 ± 3.5 years). The majority of women were between 40 and 44 years, similar to the Danish cohort where women aged ≥40 years constituted a smaller but distinct proportion of pregnancies. Educational attainment was slightly lower among older women, though not statistically significant, whereas professional employment was less frequent among AMA women. Socioeconomic disparities may partly influence pregnancy risks, as lifestyle, healthcare access, and parity differ across maternal ages [9].

Mode of delivery differed significantly between the two groups (p < 0.001), with women of AMA less likely to have spontaneous vaginal delivery and more likely to require operative delivery, consistent with previous reports that AMA is an independent predictor of non-spontaneous delivery. Physiological factors such as reduced uterine contractility and increased obstetric complications, as well as elective decisions due to perceived risks, may explain this pattern [2].

GDM was markedly higher in AMA women (48.3% vs. 14.6%), aligning with previous studies that identify AMA as a strong risk factor for impaired glucose tolerance. Age-related decline in insulin sensitivity, compounded by higher rates of obesity and metabolic syndrome, contribute to this association. Similarly, hypertensive disorders of pregnancy were more common in AMA women (11.8% vs. 2.8%), which parallels prior findings that link maternal aging to vascular stiffness and endothelial dysfunction. Pre-eclampsia was rare in our cohort (0.3%), lower than international estimates, which may reflect sample size or regional differences in comorbidity burden.

Preterm delivery (<37 weeks) was more frequent among AMA women (4.3% vs. 1.5%), supporting reports from Denmark and other large-scale cohorts that maternal age increases the risk of early delivery. However, our logistic regression analysis showed this association was not statistically significant after adjusting for confounders, suggesting that age-related preterm birth may be mediated by comorbidities rather than maternal age alone. Thyroid disorders were also more prevalent among AMA women [4], which could be attributed to autoimmune thyroid dysfunction and subclinical hypothyroidism known to rise with age [10].

Miscarriage was significantly more common in AMA pregnancies (5.3% vs. 1.2%), with logistic regression confirming AMA as an independent predictor (aOR 3.41, 95% CI 1.13-10.29). This finding is consistent with Frederiksen LE et al. [11], who demonstrated a threefold increased risk of miscarriage in women aged ≥40 years. The underlying biological mechanism is largely attributed to meiotic nondisjunction and diminished oocyte quality [4].

Congenital anomalies, including Down syndrome and multiple anomalies, were observed exclusively in AMA women in our cohort, though numbers were small and not statistically significant. This aligns with global data demonstrating that chromosomal abnormalities, particularly trisomy 21, increase sharply with advancing age [12-15]. Our findings reinforce the need for genetic counseling and early screening in AMA pregnancies.

NICU admissions were significantly higher in older mothers (3.4% vs. 0.9%, p = 0.04), reflecting the combined impact of preterm delivery, maternal comorbidities, and intrapartum complications. Neonatal mortality was rare and did not differ significantly between groups, similar to reports from Denmark where stillbirth and perinatal mortality rates were not consistently elevated among women ≥40 years [16].

Our logistic regression analysis confirmed AMA as an independent predictor of several adverse outcomes, including GDM, hypertensive disorders, cesarean delivery, miscarriage, NICU admission, and thyroid disorders. These findings are in line with Frederiksen LE et al. [11], who reported that women ≥40 years had significantly higher risks of chromosomal abnormalities, miscarriage, and preterm birth. Importantly, the persistence of associations after adjustment for parity, BMI, and socioeconomic status underscores the independent effect of maternal age.

While some studies have suggested that adequate antenatal care may mitigate risks in AMA pregnancies, our findings demonstrate that even with routine antenatal monitoring, AMA women remain at significantly elevated risk for metabolic and obstetric complications. Unlike the Danish study [17], which found no significant increase in congenital malformations, our cohort identified anomalies exclusively in the AMA group, though this was limited by small numbers. This may reflect population differences, genetic background, or under-detection in younger women due to less frequent screening [18,19].

The strengths of our study include its prospective design, inclusion of a control group, and comprehensive evaluation of both maternal and fetal outcomes in an Omani cohort. However, limitations include the relatively modest sample size compared with national registry studies, which may restrict detection of rare outcomes such as congenital anomalies or stillbirth. Additionally, lifestyle factors such as smoking and BMI, which are established confounders, were not fully captured in our cohort.

The findings of this study have important clinical implications for antenatal care in Oman and similar healthcare settings. Given the markedly increased risks of gestational diabetes, hypertensive disorders, and miscarriage in AMA pregnancies, routine screening protocols may need to be adapted for this high-risk group. Early and repeated glucose tolerance testing, blood pressure monitoring, and thyroid function assessment should be emphasized for women aged 40 years and above. In addition, genetic counseling and offering first-trimester screening for chromosomal anomalies can improve early detection and informed decision-making. Obstetricians should also anticipate higher rates of cesarean section and NICU admissions in AMA women, necessitating preparedness in terms of delivery planning and neonatal support. At the policy level, greater awareness campaigns regarding the risks associated with delayed childbearing could help women make more informed reproductive choices.

## Conclusions

In conclusion, our study demonstrates that advanced maternal age is independently associated with multiple adverse pregnancy outcomes, including gestational diabetes, hypertensive disorders, miscarriage, and increased neonatal morbidity. Although some outcomes, such as congenital anomalies and neonatal mortality, were infrequent, their occurrence exclusively among older mothers highlights the biological vulnerability associated with advanced age. Our findings align with international evidence, particularly large cohort studies, but also contribute novel regional data from Oman, where limited research has been conducted on this issue. These results underscore the importance of vigilant antenatal monitoring, individualized counseling, and targeted preventive strategies for women aged 40 years and above. Strengthening antenatal protocols and resource allocation for this group can significantly improve maternal and neonatal outcomes in the Sultanate of Oman.
